# Supplementary material for: Free and Added Sugar Consumption and Adherence to Guidelines: The UK National Diet and Nutrition Survey (2014/15–2015/16)
Source: Nutrients. 2020 Feb 1;12(2):393. doi: 10.3390/nu12020393 (PMC7071218; doi:10.3390/nu12020393)
Supplement: Supplementary file 1 [file nutrients-12-00393-s001.pdf]

Table S1. Daily intakes of added and free sugars by age and sex* (NDNS 2014-2016) (Including consumers and non-consumers).

Added and free sugars combinations		Children, 1.5-3 Years	Adults, 65+ Years		
		**Combined (*n*=250)**	**Male (*n*=141)**	**Female (*n*=194)**	**Combined (*n*=335)**
Added sugars (EFSA)[1]	g/day, Median	20.4	39.9	30.5	34.0
	g/day, P25-P75	12.3-31.5	21.7-68.0	17.3-43.0	20.2-51.5
	%TE, Median	7[a,b]	8[a,b]	8[a,b]	8[a,b]
	%TE, P25-P75	5-11	5-13	5-11	5-12
Free sugars (WHO)[2]	g/day, Median	27.0	50.4	34.4	40.6
	g/day, P25-P75	18.1-40.4	31.3-80.6	22.4-47.3	25.4-61.9
	%TE, Median	9	11	9	10
	%TE, P25-P75	6-14	7-16	6-13	6-14
Free sugars (SACN)[3]	g/day, Median	28.9	51.1	34.9	41.7
	g/day, P25-P75	18.5-42.4	31.5-81.1	22.5-47.7	25.5-62.2
	%TE, Median	10	11	9	10
	%TE, P25-P75	7-15	7-16	16-13	6-14

NDNS: UK National Diet and Nutrition Survey. n= number of participants. [1]Added sugars (European Food Safety Authority, EFSA): Sugar—table + Sugar—other; [2]Free sugars (World Health Organisation, WHO): Added sugars (EFSA) + Sugar—honey + Sugar—fruit juice; [3]Free sugars (UK Scientific Advisory Committee on Nutrition, SACN): Free sugars (WHO) + Sugar—pureed fruit; P25-P75: Inter-Quartile Range (percentile 25th–75th). [a]Significant difference between free sugars (WHO) and added sugars (EFSA) ($p < 0.05$). [b]Significant difference between free sugars (SACN) and added sugars (EFSA) ($p < 0.05$). [c]Significant difference between % Total Energy (TE) of adults and children ($p < 0.05$). [d]Significant difference between% TE of adults and teenagers ($p < 0.05$).

Table S2. Daily intakes of individual sugar components by age and sex* (NDNS 2014-2016) (Including consumers and non-consumers)

Individual sugar components		Children, 1.5-3 Years	Adults, 65+ Years		
		Combined (n=250)	Male (n=141)	Female (n=194)	Combined (n=335)
Sugar – table[1]	g/day, Median	16.6	34.4	25.2	28.4
	g/day, P25-P75	10.4-25.3	18.6-52.8	15.9-36.7	17.1-45.1
	%TE, Median	6	7	6	7
	%TE, P25-P75	4-9	4-11	4-11	4-11
Sugar – other[2]	g/day, Median	3.0	4.5	3.6	4.2
	g/day, P25-P75	1.0-5.6	2.0-7.2	1.3-5.9	1.6-6.6
	%TE, Median	1	1	1	1
	%TE, P25-P75	0-2	0-1	0-1	0-1
Sugar – honey[3]	g/day, Median	0.0	0.0	0.0	0.0
	g/day, P25-P75	0.0-0.0	0.0-0.1	0.0-0.2	0.0-0.2
	%TE, Median	0	0	0	0
	%TE, P25-P75	0-0	0-0	0-0	0-0
Sugar - fruit juice[4]	g/day, Median	2.2	5.3	0.4	1.2
	g/day, P25-P75	0.5-7.9	0.7-15.5	0.0-3.2	0.1-10.6
	%TE, Median	1	1	0	0
	%TE, P25-P75	0-3	0-3	0-1	0-2
Sugar - pureed fruit[5]	g/day, Median	0.3	0.3	0.2	0.3
	g/day, P25-P75	0.1-0.9	0.0-0.7	0.0-0.6	0.0-0.7
	%TE, Median	0	0	0	0
	%TE, P25-P75	0-0	0-0	0-0	0-0
Sugar - stewed fruit[6]	g/day, Median	0.0	0.0	0.0	0.0
	g/day, P25-P75	0.0-0.0	0.0-0.7	0.0-0.2	0.0-0.4
	%TE, Median	0	0	0	0
	%TE, P25-P75	0-0	0-0	0-0	0-0
Sugar - dried fruit[7]	g/day, Median	0.7	1.3	1.0	1.3
	g/day, P25-P75	0.0-4.5	0.0-5.7	0.0-3.8	0.0-4.5
	%TE, Median	0	0	0	0
	%TE, P25-P75	0-1	0-1	0-1	0-1

NDNS: UK National Diet and Nutrition Survey. n= number of participants. [1]Sugar—table: Including all sucrose sugars such as granulated sugar used in baking/confectionary and cakes, etc.; [2]Sugar—other: Sugar in other sugar-based sweeteners such as glucose syrup; [3]Sugar—honey: Sugar in honey; [4]Sugar—fruit juice: Sugar in fruit and vegetable juice; [5]Sugar—pureed fruit: Sugar in fruit puree; [6]Sugar—stewed fruit: Sugar in stewed fruit; [7]Sugar—dried fruit; Sugar in dried fruit; % TE: total energy. P25–P75: Inter-Quartile Range (percentile 25th–75th).

Table S3. Daily intakes of individual sugar components by age and sex* (NDNS 2014-2016) (Consumers only)

Individual sugar components		Children, 1.5-3 Years	Adults, 65+ Years		
		Combined	Male	Female	Combined
Sugar – table[1]	% Consumers	100	100	100	100
	g/day, Median	16.6	34.4	25.2	28.4
	g/day, P25-P75	10.4-25.3	18.6-52.8	15.9-36.7	17.1-45.1
	%TE, Median	6	7	6	7
	%TE, P25-P75	4-9	4-11	4-11	4-11
Sugar – other[2]	% Consumers	99	98	98	98
	g/day, Median	3.0	4.6	3.6	4.2
	g/day, P25-P75	1.0-5.6	2.0-7.2	1.5-5.9	1.7-6.7
	%TE, Median	1	1	1	1
	%TE, P25-P75	0-2	0-1	0-1	0-1
Sugar – honey[3]	% Consumers	24	26	25	26
	g/day, Median	1.0	4.0	1.6	1.8
	g/day, P25-P75	0.1-3.2	0.2-6.4	0.4-5.3	0.3-5.6
	%TE, Median	0	1	0	0
	%TE, P25-P75	0-1	0-1	0-1	0-1
Sugar - fruit juice[4]	% Consumers	94	90	84	87
	g/day, Median	3.0	7.7	1.0	2.6
	g/day, P25-P75	0.7-8.6	1.0-16.4	0.2-3.7	0.3-12.2
	%TE, Median	1	2	0	1
	%TE, P25-P75	0-3	0-3	0-1	0-2
Sugar - pureed fruit[5]	% Consumers	92	74	75	74
	g/day, Median	0.4	0.5	0.4	0.4
	g/day, P25-P75	0.2-1.0	0.3-0.9	0.2-0.7	0.2-0.8
	%TE, Median	0	0	0	0
	%TE, P25-P75	0-0	0-0	0-0	0-0
Sugar - stewed fruit[6]	% Consumers	22	48	44	46
	g/day, Median	0.2	0.6	0.4	0.5
	g/day, P25-P75	0.1-0.8	0.1-3.3	0.1-1.6	0.1-2.8
	%TE, Median	0	0	0	0
	%TE, P25-P75	0-0	0-1	0-0	0-1
Sugar - dried fruit[7]	% Consumers	55	57	61	59
	g/day, Median	3.8	5.0	3.2	3.8
	g/day, P25-P75	0.1-17.9	0.4-34.7	0.2-18.4	0.2-24.9
	%TE, Median	1	1	1	1
	%TE, P25-P75	0-6	0-9	0-4	0-5
Bases (unweighted)	n, consumers and non-consumers	250	141	194	335

NDNS: UK National Diet and Nutrition Survey. [1]Sugar—table: Including all sucrose sugars such as granulated sugar used in baking/ confectionary and cakes, etc.; [2]Sugar—other: Sugar in other sugar-based sweeteners such as glucose syrup; [3]Sugar—honey: Sugar in honey; [4]Sugar—fruit juice: Sugar in fruit and vegetable juice; [5]Sugar—pureed fruit: Sugar in fruit puree; [6]Sugar—stewed fruit: Sugar in stewed fruit; [7]Sugar—dried fruit; Sugar in dried fruit; % TE: total energy. % Consumers: Percentage of consumers of this type of sugar; P25-P75: Inter-Quartile Range (percentile 25th- 75th). n= number of participants.

Table S4. Percentage meeting recommendations for free sugars intake by age groups and sex* (NDNS 2014-2016) (Including consumers and non-consumers)

Added and free sugars combinations		Children, 1.5-3 Years	Adults, 65+ Years		
		Combined (n=250)	Male (n=141)	Female (n=194)	Combined (n=335)
Added sugars (EFSA)[1]	<5 %TE[4]	27	27	23	25
	<10 %TE[5]	70	61	66	64
Free sugars (WHO)[2]	<5 %TE[4]	15	11	18	15
	<10 %TE[5]	53	43	59	52
Free sugars (SACN)[3]	<5 %TE[4]	14	9	17	13
	<10 %TE[5]	49	42	58	51

All values are %.

NDNS: UK National Diet and Nutrition Survey. n= number of participants. [1]Added sugars (European Food Safety Authority, EFSA): Sugar—table + Sugar—other; [2]Free sugars (World Health Organisation, WHO): Added sugars (EFSA) + Sugar—honey + Sugar—fruit juice; [3]Free sugars (UK Scientific Advisory Committee on Nutrition, SACN): Free sugars (WHO) + Sugar—pureed fruit. [4]Recommendation by the SACN (<5% TE: Below 5% of total energy); [5]Recommendation by the WHO (<10% TE: Below 10% of total energy).

Table S5. Contribution (%) of NDNS RP food group sources to intake of sugars by age (1.5-3 Years) (NDNS 2014-2016)*

	Children, 1.5-3 Years (N=250)							
	**Added sugars (EFSA)[1]**	**Free sugars (WHO)[2]**	**Free sugars (SACN)[3]**	**Sugar - honey**	**Sugar - fruit juice**	**Sugar - pureed fruit**	**Sugar - stewed fruit**	**Sugar - dried fruit**
Cereals and cereal products	38.8	31.8	30.5	11.8	1.8	3.8	9.6	19.9
- Breakfast cereals	9.2	7.7	7.2	8.3	0.0	0.0	0.0	5.9
- Biscuits, buns, cakes, pastries, fruit pies	25.5	20.8	20.2	3.5	1.4	3.6	9.6	13.6
- Pasta, rice, bread and other cereals	4.1	3.3	3.1	0.1	0.4	0.3	0.0	0.4
Sugars, preserves, confectionery	21.5	20.5	20.3	12.5	7.2	13.3	0.0	3.5
- Sugars[4], preserves, sweet spreads	6.9	7.6	7.5	12.5	0.2	11.0	0.0	0.0
- Sugars consumed in tea and coffee	1.5	1.4	1.4	0.0	0.0	0.0	0.0	0.0
- Sugar confectionery	5.4	5.1	5.3	0.0	7.0	2.3	0.0	3.4
- Chocolate confectionery	7.7	6.3	6.2	0.0	0.0	0.0	0.0	0.2
Milk and milk products	25.0	20.3	19.5	1.9	2.5	35.2	1.4	0.0
- Yogurt, fromage frais, dairy desserts	14.2	11.8	11.6	1.9	0.8	33.3	1.4	0.0
- Ice cream	5.0	3.7	3.6	0.0	0.1	1.7	0.0	0.0
- Cream and other milks[5]	5.8	4.7	4.3	0.0	0.9	0.3	0.0	0.0
Fruits and fruit products	0.0	0.0	0.9	0.0	0.1	6.7	5.0	29.6
Non-alcoholic beverages	7.1	20.7	21.3	0.0	76.5	23.7	0.2	0.0
- Fruit and vegetable juice	0.2	12.2	12.4	0.0	39.7	3.5	0.0	0.0
- Soft drinks[6] (not diet[7])	6.9	6.9	6.8	0.0	10.4	0.7	0.2	0.0
- Soft drinks[6] (diet[7])	0.0	1.5	2.2	0.0	26.4	19.4	0.0	0.0
Alcoholic beverages	0.0	0.0	0.0	0.0	0.0	0.0	0.0	0.0
Miscellaneous	3.7	3.5	4.2	0.0	4.3	4.9	3.5	1.4
- Beverages dry weight[8]	1.7	1.6	1.5	0.0	0.0	0.0	0.0	0.0
- Soups	0.4	0.3	0.3	0.0	0.0	0.0	0.0	0.0
- Savoury sauces and condiments	1.4	1.1	1.0	0.0	2.0	0.1	1.1	0.0
Bases (unweighted)	250	250	250	250	250	250	250	250
% consumers	100	100	100	27	94	88	20	55

NDNS: UK National Diet and Nutrition Survey. [1]Added sugars (European Food Safety Authority, EFSA): Sugar—table + Sugar—other; [2]Free sugars (World Health Organisation, WHO): Added sugars (EFSA) + Sugar—honey + Sugar—fruit juice; [3]Free sugars (UK Scientific Advisory Committee on Nutrition, SACN): Free sugars (WHO) + Sugar—pureed fruit. [4]Discreationary sugars added to foods and drinks, apart from tea and coffee; [5]Other milks include milkshake, coffee whitener, milk drinks, hot chocolate, milk alternatives, dried milk and milks other than cows' milk. [6]Soft drinks include squashes, cordials, tonic water, energy drinks, all types of still and carbonated soft drinks and ice tea. [7]Diet refers to low-calorie, no-added-sugar (EFSA) and sugar-free varieties. [8]Beverages dry weight includes instant and powder forms of drinking chocolate, cocoa, malted drinks,

milk shake powder, tea and coffee such as cappuccino, latte and mocha with sugar. *Some food groups such as meats are removed as they don't have relevance to this table. Therefore, the sum of % contributions may not always add up to % consumers.

Table S6. Contribution (%) of NDNS RP food group sources to intake of sugars by age (65+ Years) (NDNS 2014-2016)*

	Adults, 65+ Years (N=335)							
	**Added sugars (EFSA)[1]**	**Free sugars (WHO)[2]**	**Free sugars (SACN)[3]**	**Sugar - honey**	**Sugar - fruit juice**	**Sugar - pureed fruit**	**Sugar - stewed fruit**	**Sugar - dried fruit**
Cereals and cereal products	37.8	32.0	31.7	8.2	2.4	12.1	22.3	44.3
- Breakfast cereals	5.2	4.6	4.4	8.0	0.0	0.1	0.0	14.0
- Biscuits, buns, cakes, pastries, fruit pies	28.0	23.6	23.6	0.1	2.2	11.8	21.9	29.5
- Pasta, rice, bread and other cereals	4.6	3.8	3.7	0.1	0.2	0.3	0.4	0.8
Sugars, preserves, confectionery	32.6	30.0	30.1	13.7	4.8	27.1	0.1	1.5
- Sugars[4], preserves, sweet spreads	10.9	11.1	11.4	13.7	4.0	27.0	0.1	0.0
- Sugars consumed in tea and coffee	13.7	12.4	12.3	0.0	0.0	0.0	0.0	0.0
- Sugar confectionery	1.8	1.4	1.4	0.0	0.8	0.1	0.0	0.3
- Chocolate confectionery	6.2	5.0	5.0	0.0	0.0	0.0	0.0	1.2
Milk and milk products	11.2	9.7	9.6	2.1	0.9	20.6	4.8	0.0
- Yogurt, fromage frais, dairy desserts	5.5	4.9	5.0	1.8	0.5	16.9	4.6	0.0
- Ice cream	4.7	3.9	3.8	0.2	0.2	3.7	0.0	0.0
- Cream and other milks[5]	0.9	0.9	0.8	0.0	0.2	0.0	0.0	0.0
Fruits and fruit products	1.1	0.9	1.5	0.0	0.8	3.0	16.9	12.5
Non-alcoholic beverages	7.1	13.1	13.0	0.0	35.8	3.1	0.4	0.0
- Fruit and vegetable juice	0.3	6.8	6.7	0.0	24.9	0.8	0.0	0.0
- Soft drinks[6] (not diet[7])	6.7	6.2	6.0	0.0	6.7	0.0	0.4	0.0
- Soft drinks[6] (diet[7])	0.0	0.1	0.2	0.0	3.9	2.3	0.0	0.0
Alcoholic beverages	0.2	5.6	5.5	0.0	32.0	0.7	0.0	0.0
Miscellaneous	5.1	4.3	4.3	2.9	5.0	5.3	1.7	0.3
- Beverages dry weight[8]	2.5	2.1	2.1	0.0	0.0	0.0	0.0	0.0
- Soups	0.8	0.6	0.6	0.0	0.1	0.0	0.3	0.0
- Savoury sauces and condiments	1.6	1.4	1.4	2.9	4.8	5.3	1.5	0.3
Bases (unweighted)	335	335	335	335	335	335	335	335
% consumers	100	100	100	29	87	72	48	60

NDNS: UK National Diet and Nutrition Survey. [1]Added sugars (European Food Safety Authority, EFSA): Sugar—table + Sugar—other; [2]Free sugars (World Health Organisation, WHO): Added sugars (EFSA) + Sugar—

honey + Sugar—fruit juice; [3]Free sugars (UK Scientific Advisory Committee on Nutrition, SACN): Free sugars (WHO) + Sugar—pureed fruit. [4]Discretionary sugars added to foods and drinks, apart from tea and coffee; [5]Other milks include milkshake, coffee whitener, milk drinks, hot chocolate, milk alternatives, dried milk and milks other than cows' milk. [6]Soft drinks include squashes, cordials, tonic water, energy drinks, all types of still and carbonated soft drinks and ice tea. [7]Diet refers to low-calorie, no-added-sugar (EFSA) and sugar-free varieties. [8]Beverages dry weight includes instant and powder forms of drinking chocolate, cocoa, malted drinks, milk shake powder, tea and coffee such as cappuccino, latte and mocha with sugar. *Some food groups such as meats are removed as they don't have relevance to this table. Therefore, the sum of % contributions may not always add up to % consumers.
